# Creation and validation of an I-123 FP-CIT template for statistical image analysis using high-resolution SPECT for parkinsonian patients

**DOI:** 10.1007/s12149-016-1085-8

**Published:** 2016-05-25

**Authors:** Kosuke Hida, Masanari Nonokuma, Yasuo Kuwabara, Tomonobu Tani, Koichi Takano, Kengo Yoshimitsu

**Affiliations:** Department of Radiology, Fukuoka University Hospital, 7-45-1 Nanakuma, Jonan-ku, Fukuoka, 814-0180 Japan

**Keywords:** Dopamine transporter, SPECT, Statistical parametric mapping, Template, I-123 FP-CIT

## Abstract

**Objective:**

The aim of this study was to create a new template for the anatomical normalization of I-123 FP-CIT SPECT images of Japanese people to evaluate dopamine transporter binding.

**Methods:**

The subjects consisted of 16 normal control subjects (5 females and 11 males; mean age ± SD, 51.6 ± 9.5 years, ranging from 25 to 62 years) and 21 parkinsonian patients (7 females and 14 males; mean age ± SD, 70.7 ± 9.4 years, ranging from 49 to 85 years). All normal control subjects and 21 patients with parkinsonism underwent MRI. A total of 148 MBq of I-123 FP-CIT was intravenously injected as a bolus, and a SPECT scan was started 4 h later. Data were analyzed with the Statistical Parametric Mapping 8 (SPM8) software. At first, I-123 FP-CIT SPECT images were co-registered to MRI images and MRI images were normalized to Montreal Neurological Institute (MNI) space using a gray.nii template. Co-registered I-123 FP-CIT SPECT images were normalized using the predetermined normalization parameters for MRI images. Then, anatomically normalized I-123 FP-CIT SPECT images were divided by background counts individually measured using ROIs set on the cerebral cortices. The I-123 FP-CIT template was created by averaging the normalized SPECT images of the 16 normal control subjects. Thereafter, the averaged MRI images of the 16 normal control subjects were also created.

**Results:**

A visual inspection revealed that there were no apparent differences between the I-123 FP-CIT images subjected to the two methods of anatomical normalization in normal control subjects. However, a group comparison by a paired *t* test using SPM8 revealed that the I-123 FP-CIT binding was significantly higher in the substriatal and temporal regions in I-123 FP-CIT images directly normalized with the I-123 FP-CIT template than in those normalized by the predetermined parameters with MRI, while it was higher in the bilateral frontal cortical regions in the latter than in the former images.

**Conclusion:**

We successfully created an I-123 FP-CIT template for Japanese people. This template is thought to be useful and reliable for the statistical analysis of I-123 FP-CIT images, although some problems exist in the evaluation of parkinsonian patients. The results of a paired *t* test using SPM suggest that we should use the same normalization method in statistical image analyses.

## Introduction

I-123 FP-CIT (I-123 Ioflupane, DaTSCAN^®^) is a ligand for evaluating striatal dopamine transporter imaging that is mainly localized in the presynaptic dopaminergic neurons of the striatum, which consists of the caudate nucleus and the putamen [1, 2]. The striatal level of dopamine transporters decreases in Parkinson’s disease or dementia with Lewy bodies, while it is preserved in essential tremor or vascular parkinsonism [3, 4]. Therefore, I-123 FP-CIT is widely used for the differential diagnosis of dementia and parkinsonism [5]. The striatum is a relatively small structure, and is easily rotated by mispositioning or a patient’s movement. For this reason, setting ROIs manually may cause interobserver error [6]. The anatomical normalization of I-123 FP-CIT SPECT images can correct such misregistration and makes it easy to interpret the images. In addition, quantitative information, such as the specific to non-specific binding ratio, is easily obtained using ROIs set on anatomically normalized SPECT images [6]. Statistical Parametric Mapping (SPM) (Wellcome Department of Imaging Neuroscience, Institute of Neurology, University College London) is one of the most reliable softwares for a voxel-based statistical image analysis [7]. In the SPM analysis, a specific template for each ligand is needed for the anatomical normalization of the brain images [8]. However, a template for I-123 FP-CIT has not yet been prepared in SPM. Thus, we created a new template using I-123 FP-CIT and a high-resolution SPECT device for Japanese people, and compared the results between two normalization techniques, namely, FP-CIT template-based and MRI-based methods.

## Subjects and methods

The subjects consisted of 16 normal control subjects (5 females and 11 males; mean age ± SD, 51.6 ± 9.5 years, ranging from 25 to 62 years) and 21 parkinsonian patients (7 females and 14 males; mean age ± SD, 70.7 ± 9.4 years, ranging from 49 to 85 years). All of the normal control subjects were medical staff members who underwent both I-123 FP-CIT SPECT and MRI studies, and none of them showed either any neurological symptoms or an organic brain lesions, such as cerebral infarct on MRI, although three of them were found to have small white matter lesions. The parkinsonian patients were retrospectively selected, and they all underwent both I-123 FP-CIT SPECT and MRI studies, while including 11 patients with Parkinson’s disease, 3 patients with multisystem atrophy, 2 patients with secondary parkinsonism, 2 patients with progressive supranuclear palsy, 1 patient with dementia with Lewy body disease and 2 patients with parkinsonism of an unknown etiology.

In the SPECT studies, 148 MBq of I-123 FP-CIT was intravenously injected for about 20 s, and the SPECT scan was started 4 h later using a three-head gamma camera (Toshiba GCA-9300R), with a high-resolution fan beam collimator. The intrinsic and system resolution were 3.8 mm and 9.0 mm in FWHM, respectively. Data were collected for 20 min in a 128 × 128 matrix (120° rotation, 30 views, 5 s per projection), and then were reconstructed using a 3D-OSEM algorithm (iteration 4 and subset 15) without scatter correction. Attenuation correction was performed using a *μ* value of 0.06. The order and cut-off frequency of the Butterworth filter were 4 and 0.13 cycles, respectively.

MRI studies were performed using Intera Achieva (1.5 T, Phillips Co. Ltd.) or Discovery MR750w (3 T, GE Co. Ltd.). Three-dimensional brain images were obtained in the sagittal plain of a 240-mm field of view in a 256 × 256 matrix in both machines, and reconstructed for a voxel size of 0.75 × 0.94 × 0.94 mm^3^ on 1.5 T and 1.20 × 0.94 × 0.94 mm^3^ on 3 T, respectively.

**Fig. 1:**
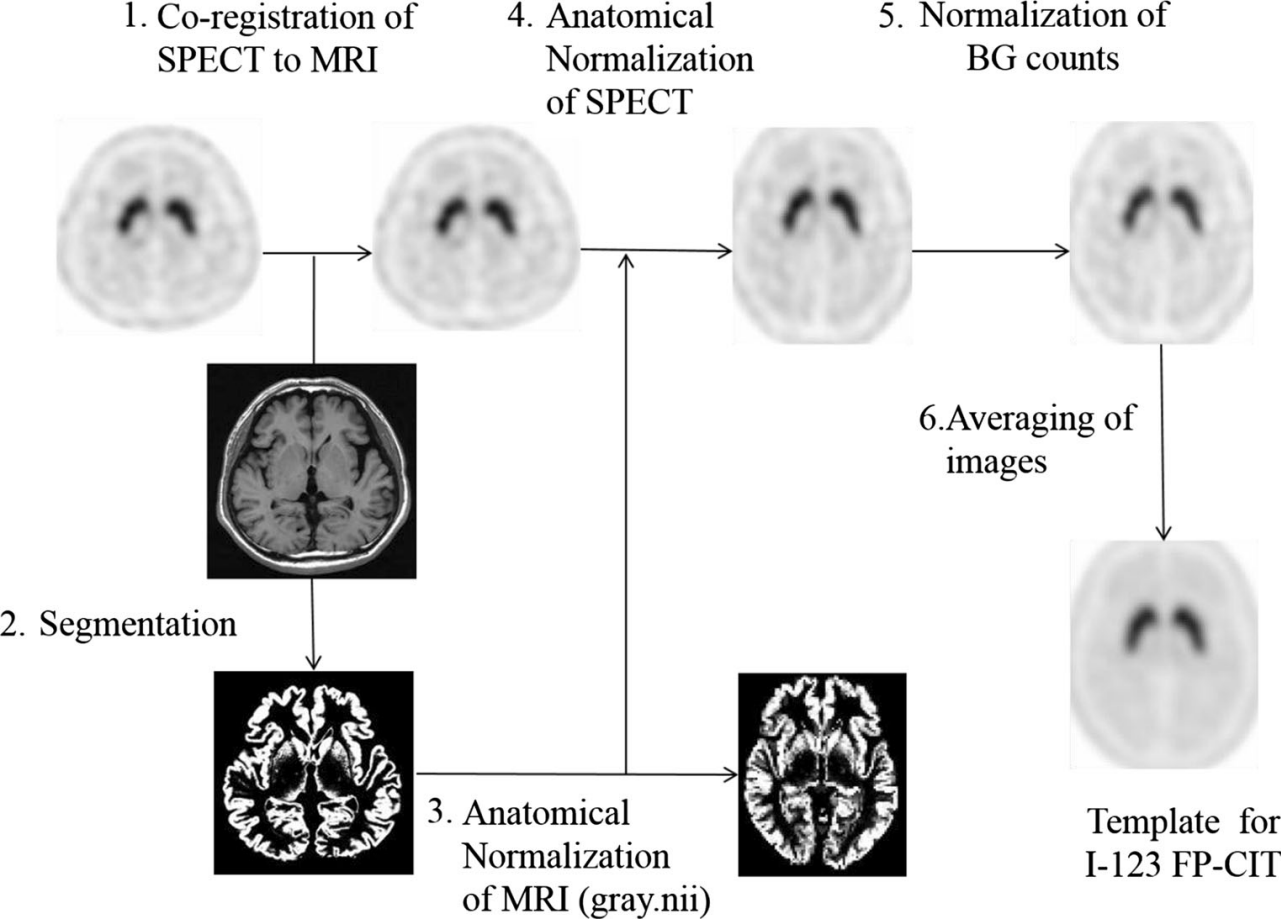
Method of creating an I-123 FP-CIT template using MRI

**Fig. 2:**
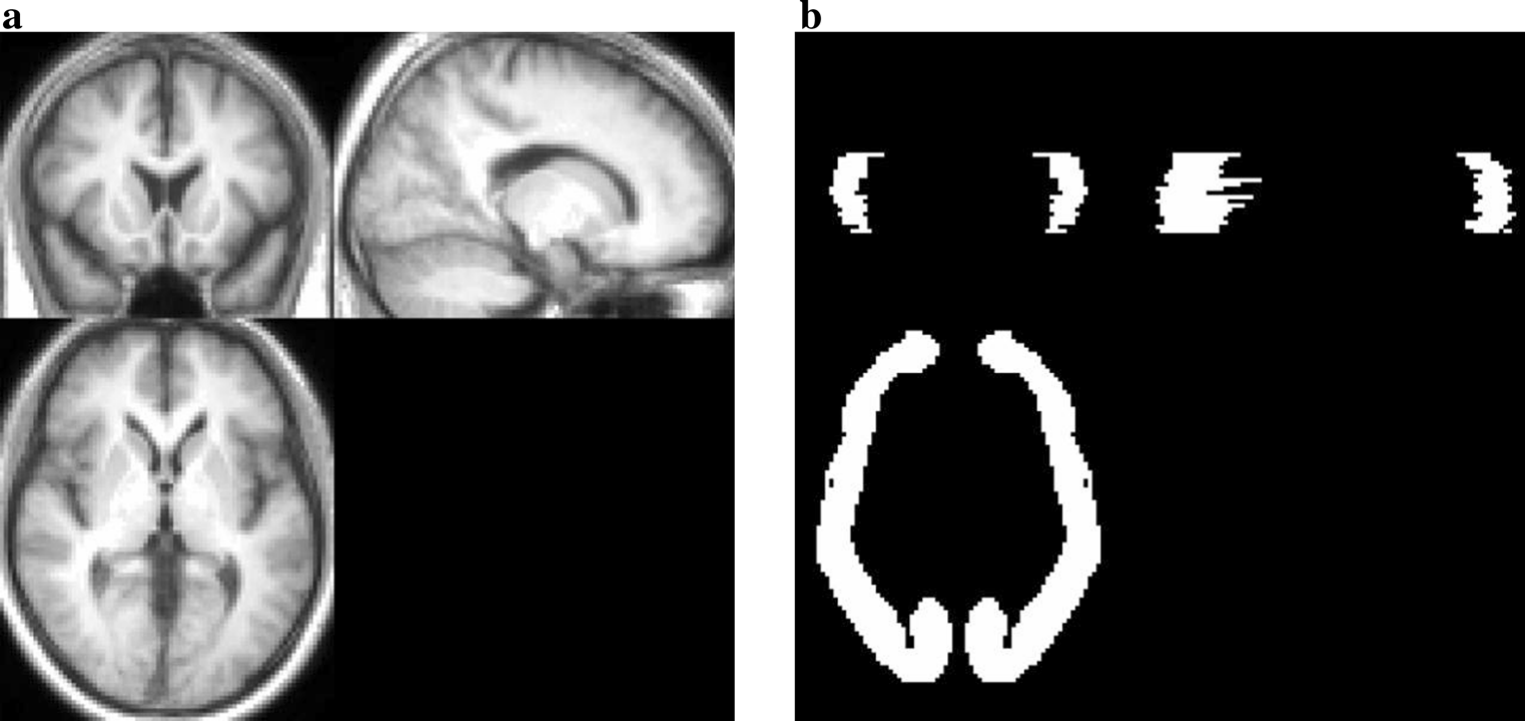
Three-dimensional T1-WI (**a**) and ROI (**b**)

Prior to the data analyses, both SPECT and MRI images were converted to the analyze format from the DICOM format using the MRIcro software, and then the sagittal MRI images were rearranged in the axial plain. A statistical image analysis was performed with SPM8 according to the method shown in Fig. 1. I-123 FP-CIT images were initially co-registered and resliced to MRI images. To determine the parameters for the anatomical normalization, MRI images were segmented into the gray and white matter, and then the gray matter images were normalized to the grey.nii template. The I-123 FP-CIT images co-registered to the corresponding MRI images were also normalized using the individually predetermined normalization parameters. After that, the radioactivity counts of the I-123 FP-CIT images were normalized using the background counts measured using three-dimensional ROIs set on cortical areas of the MRI images in order to measure the non-specific binding of I-123 FP-CIT (Fig. 2). The I-123 FP-CIT template was created by averaging the images of 16 normal control subjects. Finally, all original I-123 FP-CIT images were anatomically normalized using the I-123 FP-CIT template. To validate this direct normalization using the I-123 FP-CIT template, we compared it with the normalization by predetermined parameters with MRI using both the visual inspection of superimposed I-123 FP-CIT images on MRI and the statistical image analysis using SPM8.

The study protocol was approved by the ethics committee/institutional review board of our hospital.

## Results

**Fig. 3:**
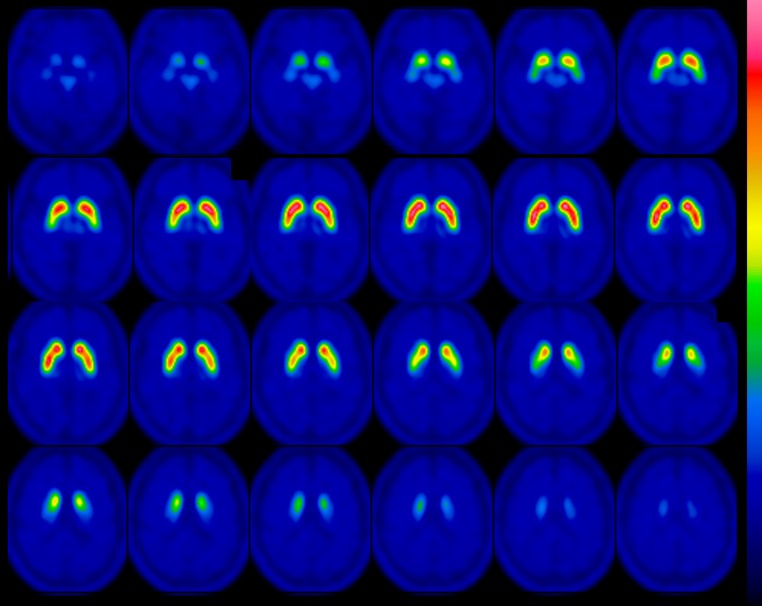
I-123 FP-CIT template images created from 16 normal control subjects

**Fig. 4:**
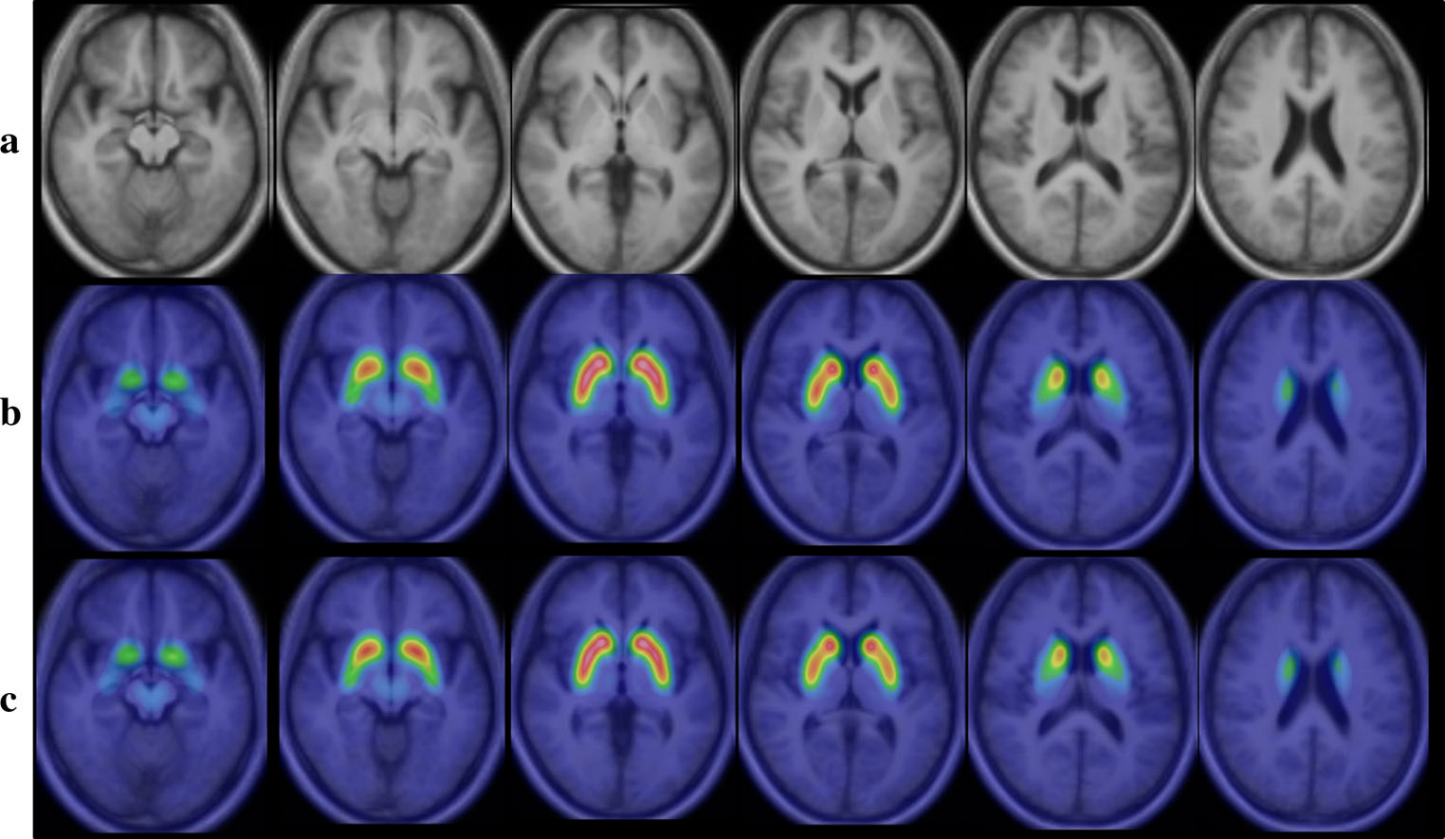
Averaged MRI images of normal control subjects (**a**), I-123 FP-CIT images normalized by the MRI-based method (**b**) and I-123 FP-CIT images directly normalized by the FP-CIT template-based method (**c**). The latter two sets of I-123 FP-CIT images are displayed superimposed on MRI images

**Fig. 5:**
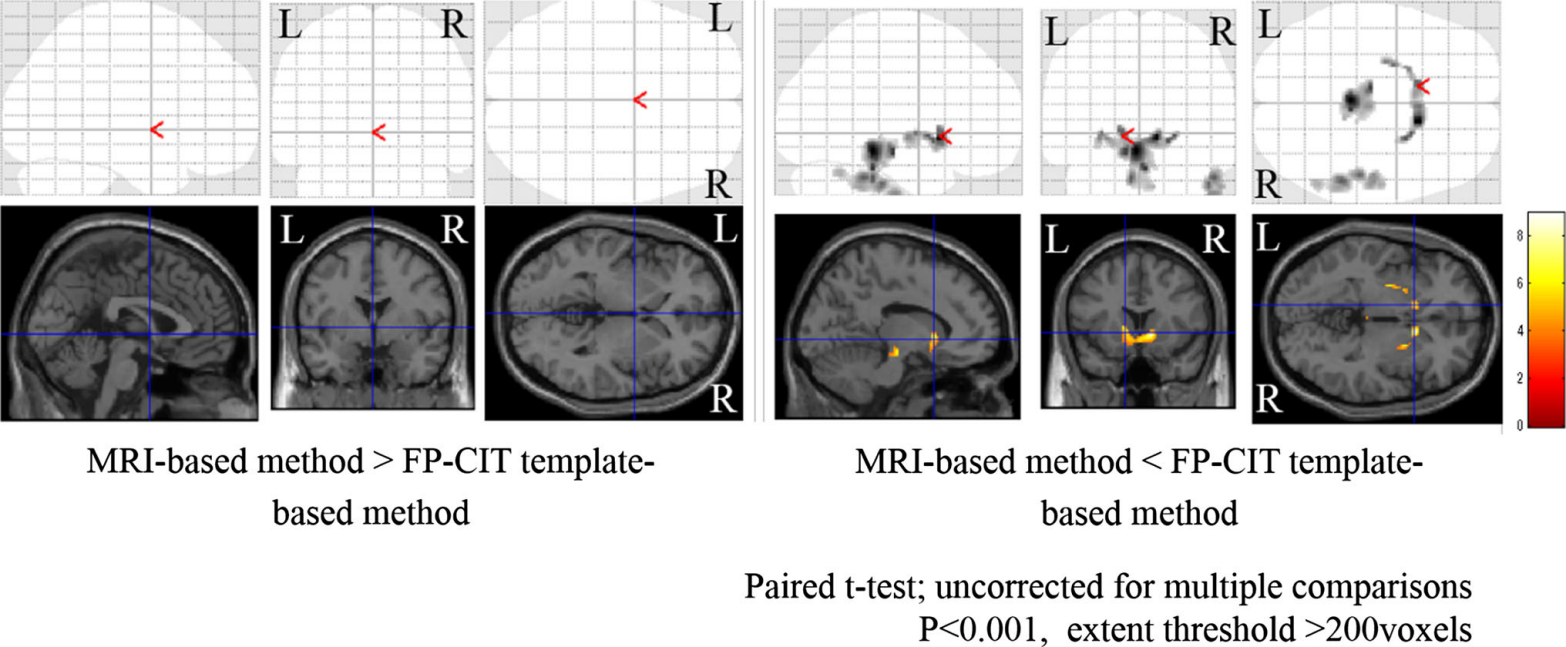
The results of a group comparison by a paired *t* test between averaged I-123 FP-CIT images anatomically normalized by the MRI-based method and the FP-CIT template-based method

**Fig. 6:**
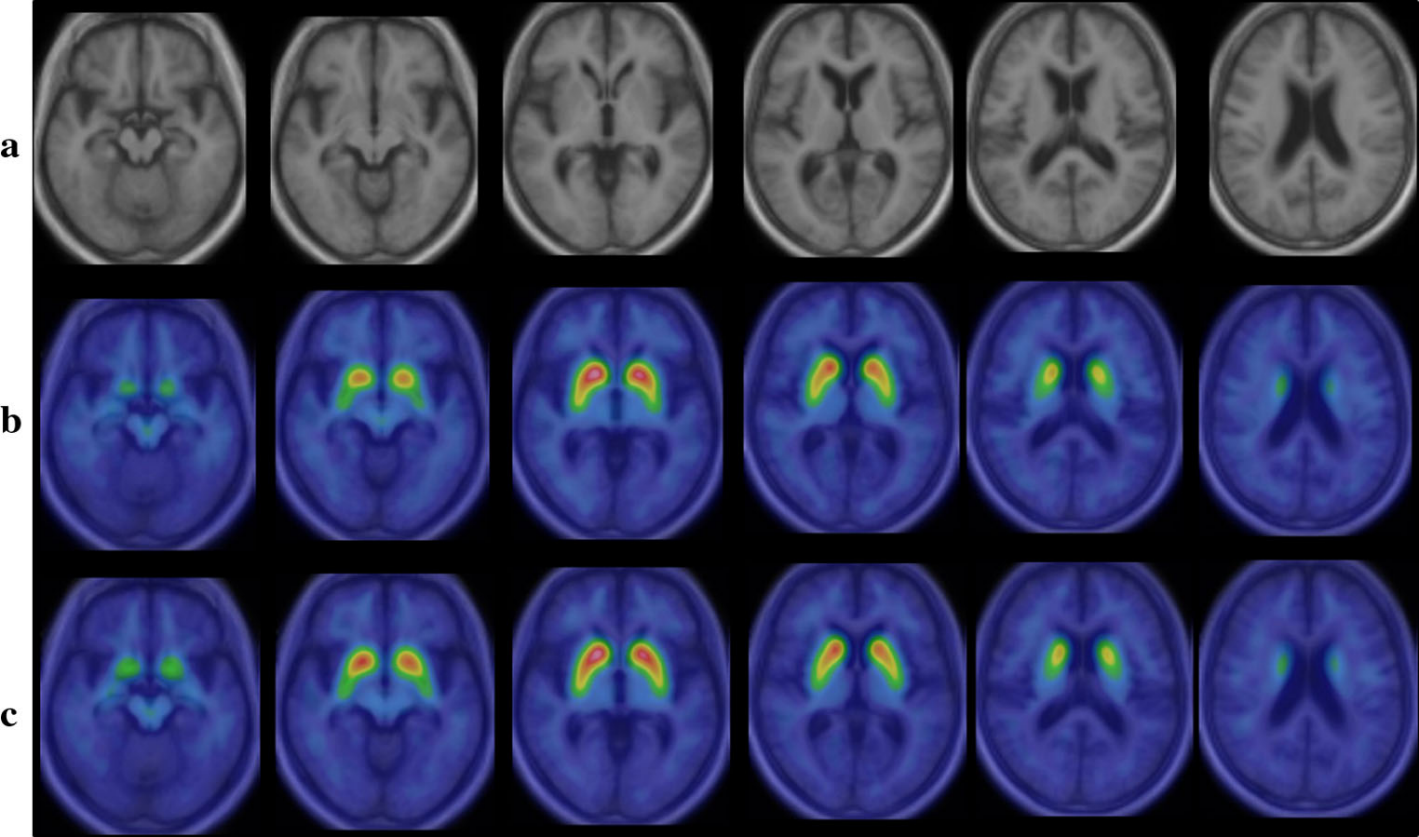
Averaged MRI images of parkinsonian patients (**a**), I-123 FP-CIT images normalized by the MRI-based method (**b**) and I-123 FP-CIT images directly normalized by the FP-CIT template-based method (**c**). The latter two sets of I-123 FP-CIT images are displayed superimposed on MRI images of parkinsonian patients

**Fig. 7:**
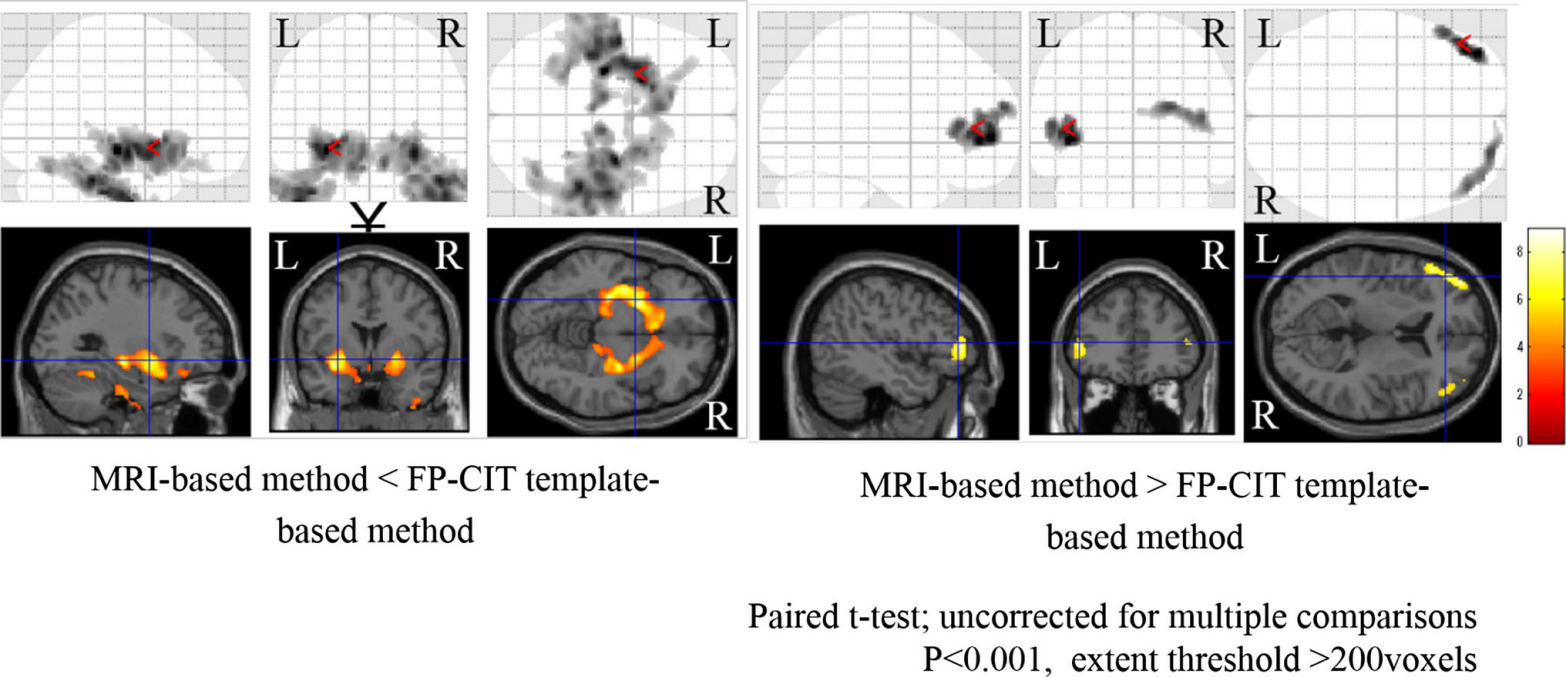
The results of a group comparison of parkinsonian patients by a paired *t* test between averaged I-123 FP-CIT images anatomically normalized by the MRI-based method and the FP-CIT template-based method

I-123 FP-CIT template images are shown in Fig. 3. Figure 4 shows averaged MRI images of normal control subjects, I-123 FP-CIT images anatomically normalized by predetermined parameters with MRI gray matter images (MRI-based method) and I-123 FP-CIT images directly normalized with the I-123 FP-CIT template (FP-CIT template-based method). The latter two sets of I-123 FP-CIT images are displayed superimposed on MRI images. A visual inspection revealed that these superimposed I-123 FP-CIT images were well fitted to the MRI images, and there was no apparent difference between them. Figure 5 shows the results of a group comparison by a paired *t* test between the averaged I-123 FP-CIT images anatomically normalized by the MRI-based method and the I-123 FP-CIT images directly normalized by the FP-CIT template-based method. There were small differences between the two normalization methods in the striatal, brain stem and cerebellar regions. Figure 6 shows averaged MRI images of parkinsonian patients, I-123 FP-CIT images anatomically normalized by the MRI-based method and I-123 FP-CIT images directly normalized by the FP-CIT template-based method. The latter two sets of I-123 FP-CIT images are displayed superimposed on MRI images of parkinsonian patients. A visual inspection revealed that the striatal radioactivity slightly shifted to the bottom of the brain in the FP-CIT template-based method compared to that in the MRI-based method. The results of a group comparison by a paired t test are shown in Fig. 7. The I-123 FP-CIT binding was significantly higher in the sub-striatal and temporal regions in I-123 FP-CIT images directly normalized by the FP-CIT template-based method than in those normalized by the MRI-based method, while it was higher in the bilateral frontal cortical regions in the latter than in the former.

## Discussion

The anatomical normalization of brain images is very useful for the clinical practice in nuclear medicine as well as brain research. SPM is one of the most widely used software for statistical image analyses [6, 10]. However, a template for I-123 FP-CIT has not yet been prepared in SPM. Thus, we created a new template for a statistical image analysis of I-123 FP-CIT images using SPM8 and high-resolution SPECT. To the best of our knowledge, there have been no reports on an I-123 FP-CIT template for Japanese people.

I-123 FP-CIT accumulates mainly in the striatum, which is the specific binding site of the dopamine transporter, while the radioactivity of non-specific binding sites, such as cerebral cortical regions, is very low [11, 12]. Thus, it is expected that there are some difficulties in the co-registration of I-123 FP-CIT images to MRI.

There have been several studies on the creation of templates for I-123 FP-CIT. Kas et al. [9] used SPM99 and an MNI-based template of ^11^C-raclopride for normalization because of its high specific striatal binding and low cortical uptake, which is similar to the profile of I-123 FP-CIT images. Garcia-Gómez et al. [13] normalized I-123 FP-CIT images using the template for magnetic resonance in T1-weighted sequences available in SPM5. The updated templates are open on the website by this group (http://www.nitrc.org/projects/spmtemplates), and can be used for SPM analysis of F-18 DOPA PET and I-123 FP-CIT SPECT. Although the spatial resolution is lower than that of our template. Colloby et al. [14] constructed a template by averaging 33 subjects and transformed them into the standard MNI space by multimodality co-registration to a high-resolution T1 MR image in standard MNI space. However, these studies directly normalized I-123 FP CIT images using templates for other tracers, and then created a template specific for I-123 FP-CIT.

In the present study, we created a template by using MRI-aided spatial normalization. This technique has been applied to a F-18 FDG template by Gispert et al. [15] and a C-11-raclopride template by Meyer et al. [16]. Although MRI-aided spatial normalization requires MRI images of each subject for the creation of a template, this method is considered more accurate than the one performed by using only functional images [15, 16]. In the present study, we could accurately co-register I-123 FP-CIT images to MRI using SPM8, as shown in Fig. 3. There were no subjects in whom the co-registration failed. However, in our experience, SPM2 sometimes failed in the anatomical normalization (data not shown), as described elsewhere [9].

In addition, we revealed a difference in the results between the two anatomical normalization methods using SPM8. The use of an I-123 FP-CIT template makes it easy to normalize the individual I-123 FP-CIT to MNI space without co-registration to MRI. As shown in Fig. 4, a visual inspection of I-123 FP-CIT images superimposed on MRI images demonstrated no apparent difference between the two normalization methods in normal control subjects. However, the striatal radioactivity slightly shifted to the bottom of the brain in the FP-CIT template-based method compared to that in the MRI-based method in the group of parkinsonian patients (Fig. 6). In addition, an SPM analysis by a paired *t* test revealed a significant difference between the two normalization methods, especially in the group of parkinsonian patients. The reason for this difference is speculated to be that a minimal co-registration or normalization error affected the results. In addition, cerebral atrophy may cause a normalization error, which is more likely in the case of parkinsonian patients who show mild cerebral atrophy on MRI. In the group of parkinsonian patients, the mean age was higher than in the normal control group. As the striatal dopamine transporters decrease with aging [17], the I-123 FP-CIT template would ideally be created using age-matched control subjects. However, it is challenging to create templates for different age groups, and the use of various templates may cause an error in the results of SPM analyses. The results of this study are thought to be reasonable, and suggest that we should use the same normalization method in SPM analyses.

## Conclusion

We successfully created an I-123 FP-CIT template for Japanese people. This template is thought to be useful and reliable for the statistical image analysis of I-123 FP-CIT images, although some problems exist in the evaluation of parkinsonian patients.
